# Temporal transcriptomics uncover dynamic interactions between pathogenic *Escherichia coli* and phage vB_Eco_K1B4

**DOI:** 10.3389/fmicb.2025.1668341

**Published:** 2025-11-11

**Authors:** Guoliang Wang, Xin Liu, Junjun Qin, Yunhan Wang, Bingzhen Ji, Jing Sun, Yanqiang Wang, Lin Zhang, Lili Zhang, Chunhui Lulong, Miao Cai, Yunxia Zhang, Yingxiang Hong, Hongxia Qiao, Xiaoqin Wang, Pengfei Gao, Guiming Liu

**Affiliations:** 1 Beijing Key Laboratory of Agricultural Genetic Resources and Biotechnology, Institute of Biotechnology, Beijing Academy of Agriculture and Forestry Sciences, Beijing, China; 2 Key Laboratory for Northern Urban Agriculture Ministry of Agriculture and Rural Affairs, Beijing University of Agriculture, Beijing, China; 3 College of Animal Science, Shanxi Agricultural University, Taigu, China

**Keywords:** *Escherichia coli*, temporal RNA-Seq, bacteriophage, bacteria-phage interaction, phage therapy

## Abstract

Pathogenic *Escherichia coli* has a serious impact on animal husbandry. Currently, people mainly prevent pathogenic bacteria by injecting antibiotics into livestock. However, such frequent use of antibiotics accelerates the development of bacterial resistance and affects people’s health. Using bacteriophages to hunt down pathogenic bacteria has become an efficient method. In this study, we identified and characterized the K1 capsular vB_Eco_K1B4 bacteriophage and used RNA-seq analysis to profile the phage transcripts during the *E. coli* infection phase. The experimental results showed that bacteriophage vB_Eco_K1B4 still had a survival rate of over 50% in a 70 °C water bath for 1 h, and could survive for a short period at low temperatures. Not only that, vB_Eco_K1B4 also has a high tolerance for relatively extreme pH environments, so this bacteriophage has the potential to inhibit pathogenic bacteria. This study further explores the transcriptional regulation mechanism during the interaction between bacteriophages and hosts. Differentially expressed genes analysis, GO enrichment, and other analysis results show that vB_Eco_K1B4 can accurately regulate the host’s transcriptional resources, while inhibiting the expression of genes related to host structural component formation and upregulating the expression of genes related to host energy metabolism. Moreover, vB_Eco_K1B4 also impacts the host’s defense mechanism against bacteriophages. These transcriptome data provide a more thorough understanding of the cellular response of *E. coli* to phage infection and aid in understanding the phage-host interaction at the transcriptomic level.

## Highlights

- Isolation of bacteriophage vB_Eco_K1B4 for K1 capsule *E. coli* B4.
- Genomic characterization of both vB_Eco_K1B4 and *E. coli* K1B4.
- Time-series transcriptomics between pathogenic *E. coli* and phage vB_Eco_K1B4.

## 1 Introduction

*Escherichia coli* is one of the commonest opportunistic pathogens in animal husbandry, which can cause economic loss. Antibiotic treatments may be the best choice against bacteria. However, using antibiotics frequently leads to acquire resistance. Many pathogens obtained resistance against many kinds of antibiotics. Global action plan on antimicrobial resistance (2015) released by World Health Organization (WHO) and the National Action Plan for the Reduction of Veterinary Antimicrobial Drug Use (2021–2025) released by China’s Ministry of Agriculture and Rural Affairs aim to effectively against abusing antibiotics. Antibiotic resistance in bacterial pathogens presents a substantial threat to the control of infectious diseases. The European Commission announced a five-year action plan to combat bacterial resistance in 2011 to ensure proper antibiotic use in humans and livestock and improve veterinary antibiotic monitoring. Animals in the U.S. are given a lot of antibiotics to prevent bacterial infections, not because they already have them (Golkar et al., 2014). Although the government has issued relevant regulations since 2018 to caution citizens against abusing antibiotics, the overall state of affairs still exists.

Due to the increase in drug resistance of microbial pathogens, the focus on solving the problem of bacterial drug resistance is gradually shifting to bacteriophage therapy (Chegini et al., 2020; Chegini et al., 2021; Ibrahim et al., 2021; Bolsan et al., 2024). The bacteriophage has been the most prevalent and widely distributed community of organisms in varied settings, from high-altitude habitats to deep-sea sediments (Adriaenssens et al., 2017; Jian et al., 2021). Unlike antibiotics, phages can only attack the target bacteria without disrupting the surrounding flora instead of killing all the bacteria (Zhang et al., 2022; Liang et al., 2023). Phage therapy may re-enter the spotlight and become widely used as an alternative Multi-Drug Resistance (MDR) therapy method (Luong et al., 2020; Kaur et al., 2021). Because the phage generated more phage particles when it entered the bacterial lysis cell, like self-amplification, it was more effective than an antibiotic (Kortright et al., 2019). Many studies have shown that bacteriophage therapy successfully treated animals, such as calves, lambs, and pigs (Smith and Huggins, 1983; Jamal et al., 2018; Desiree et al., 2021). Compared with conventional antibiotic treatments, phage therapy has several potential advantages. There are more natural phages available due to extreme biodiversity. As previously mentioned, a number of high-profile phage genomes and characteristics have been proved, coupled with more widely available technology for phage identification and production, and have led to more widespread use of phages in clinical medicine over the past several years. Besides, each bacteriophage attacks only a very limited number of bacteria and is almost specific to one type of bacteria, so they can target diseasespecific bacteria without affecting the normal flora in the host. Bacteriophages are “living drugs” that increase in number as the target bacterial population spreads, so they can be administered in small doses.

Among pathogenic *E. coli* strains the K1 capsular type is predominant, causing various animal diseases and human infections (Scholl et al., 2001; Stummeyer et al., 2006; Antoine et al., 2023). The capsule is a significant virulence factor and crucial antigen in *E. coli* K1 (Scholl et al., 2005; Moller-Olsen et al., 2020), facilitating bacterial survival and proliferation both within and outside the digestive system. Despite the isolation and genomic proteomic characterization of numerous K1 phages (Santos et al., 2011; Gong et al., 2021), comprehensive transcriptomic profiling of bacteria-phage interactions remains limited. Improved understanding of phage transcriptomes is necessary for several reasons, which include temporal regulation of phage gene expression during an infection, a frequently observed concomitant usurpation of the host’s gene expression machinery, and a paucity of functional knowledge for the vast majority of phage genes.

Genome-wide analysis of transcripts using RNA-Seq is a convincing way to illuminate the differential expression of bacterial gene signatures under different conditions (Blasdel et al., 2018). In this study, we mainly focused on changes of both host and phage genes after phage vB_Eco_K1B4 infection with *E. coli*. Our understanding of phage therapeutic features may benefit from investigating temporal changes in bacteria-phage interaction.

## 2 Materials and methods

### 2.1 Bacterial strain, growth conditions and bacteriophage isolation

This study used the K1 capsule *E. coli* B4 isolated from pig feces as host bacteria. Briefly, pig feces samples held at −80 °C were thawed in a 50 mL centrifuge tube, then 5 g of material was taken, and 40 mL of SM buffer was added (NaCl 5.8 g, MgSO4 7H2O 2.0 g, 1 mol/L Tris HCl 50 mL pH7.0, gelatin 0.1 g). The bacteriophage samples suspension was vortexed for 5 min, then incubated in a shaker for 30 min before being chilled for 5 min. The supernatant was filtered twice using a sterile Millipore membrane syringe filter (0.22 μm, Millex Syringe-driven Filter Unit, Sigma-Aldrich) after being centrifuged at 5,000 g for 10 min. Plaques were seen on the LB plates after the filtrate had been spotted on them and incubated overnight at 37 °C.

### 2.2 Whole genome sequencing of *Escherichia coli* K1B4 and vB_Eco_K1B4 capsule

The genomic DNA of host bacteria was extracted and purified with Bacteria Genomic DNA kit (CWBIO, Cat. CW05525S). Subsequently, gDNA was fragmented by Covaris M220, and VAHTS Universal DNA Library Prep Kit performed the sequencing library for Illumina (Vazyme) following the manufacture. Paired-end sequencing was performed on Illumina Novaseq 6000 (Novegene).

### 2.3 Purification, propagation and transmission electron microscopy (TEM) of the phage vB_Eco_K1B4

*Escherichia coli* K1 was grown in LB medium at 37 °C and 180 rpm for 12–18 h, without any supplementation. The prepared phages were then deposited to carboncoated copper grids for negative staining, and examined utilizing a transmission electron microscope (Thermo Fisher Talos L120C) with a voltage of 120 kV.

### 2.4 One-step growth curve determination

The one-step growth curve of K1_SXAU_B4 was determined by referring to the method of Ellis et al. Phage and the host bacteria strain were taken 750 μL respectively, and mixed according to the optimal MOI, incubated in a 37 °C water bath for 15 min, then centrifuged at 10,000 g for 3 min and discarded supernatant. The precipitate was then washed twice with 0.5 mL LB liquid medium to remove the phage that not adsorbed on the bacteria, centrifuged at 10,000 g for 3 min, followed by resuspension of the sediment with pre-warmed 5 mL LB liquid medium at 37 °C, and incubated rapidly on a 37 °C shaker with 200 rpm.

### 2.5 RNA-seq library construction and sequencing

Total RNA was extracted from each sample using TRIzol reagent according to the manufacturer’s instructions (CWBIO, Cat.0581 M). The rRNA depletion was performed by Ribo-off rRNA Depletion Kit (Vazyme, N407) according to the manufacturers. The ribo-off RNA samples were purified with 2x VAHTS RNA Clean Beads (Vazyme, N412), and then the RNA-seq library was used VAHTS Universal V8 RNA-seq Library Prep Kit for Illumina following the protocol. The concentration of library product was measured by Qubit3.0 (Invitrogen) and the size distribution was checked by gel electrophoresis. Libraries were sequenced on the Illumina Novaseq 6,000 system at the Novagene.

### 2.6 RNA-seq analysis

Raw transcriptome sequencing data were quality-controlled and evaluated using Fastp (version 0.23.4) (Chen, 2023). The transcriptome library after each quality control was aligned to the combined transcripts of *E. coli* K1B4 and bacteriophage vB_Eco_K1B4 using Hisat2 (version 2.2.1) (Kim et al., 2019). Samtools (version 1.6) (Danecek et al., 2021) was used to sort the sam files in comparison. HTseq-count (version 2.0.5) and Stringtie (version 2.1.7) (Pertea et al., 2016) were then used to calculate the count and FPKM values, respectively. DESeq2 (Love et al., 2014) was used to calculate the differential genes with 0 min as the control, and the statistical standard was padj<0.05, and the differential fold (FC) was ≥ 2-fold or ≤ − 2-fold. Cytoscape (version 3.10.2) (Shannon et al., 2003) was used for visualization of coexpression network analysis.

### 2.7 Real-time quantitative PCR of DEGs

To validate RNA-seq data, real-time quantitative PCR (RT-qPCR) was performed on 8 selected differential expression genes (DEGs). All the primers for RT-qPCR were listed on the blow. The cDNA strand was synthesized using HiScript III 1st Strand cDNA Synthesis Kit (+gDNA wiper) (Vazyme, R312). The Actin gene was used as an internal control to normalize the expression level, and all the reactions were performed in triplicate. The RT-qPCR reactions were as followed: 10 μL 2x AceQ Universal SYBR qPCR Master Mix (Vazyme, Q511), forward and reverse primers were added 0.4 μL, diluted template cDNA 2 μL, and nuclease-free water was up to 20 μL. The RT-qPCR reactions were performed on the CFX96 system (Bio-Rad) and the PCR reactions program as followed: 95 °C 5 min, 40 cycles of 95 °C 10s, 60 °C 30s. The fold change of the expression target gene was calculated using the comparative Ct method with formula 2^-ΔΔCT^.

## 3 Results

### 3.1 Isolation and identification of phage vB_Eco_K1B4

**Figure 1:**
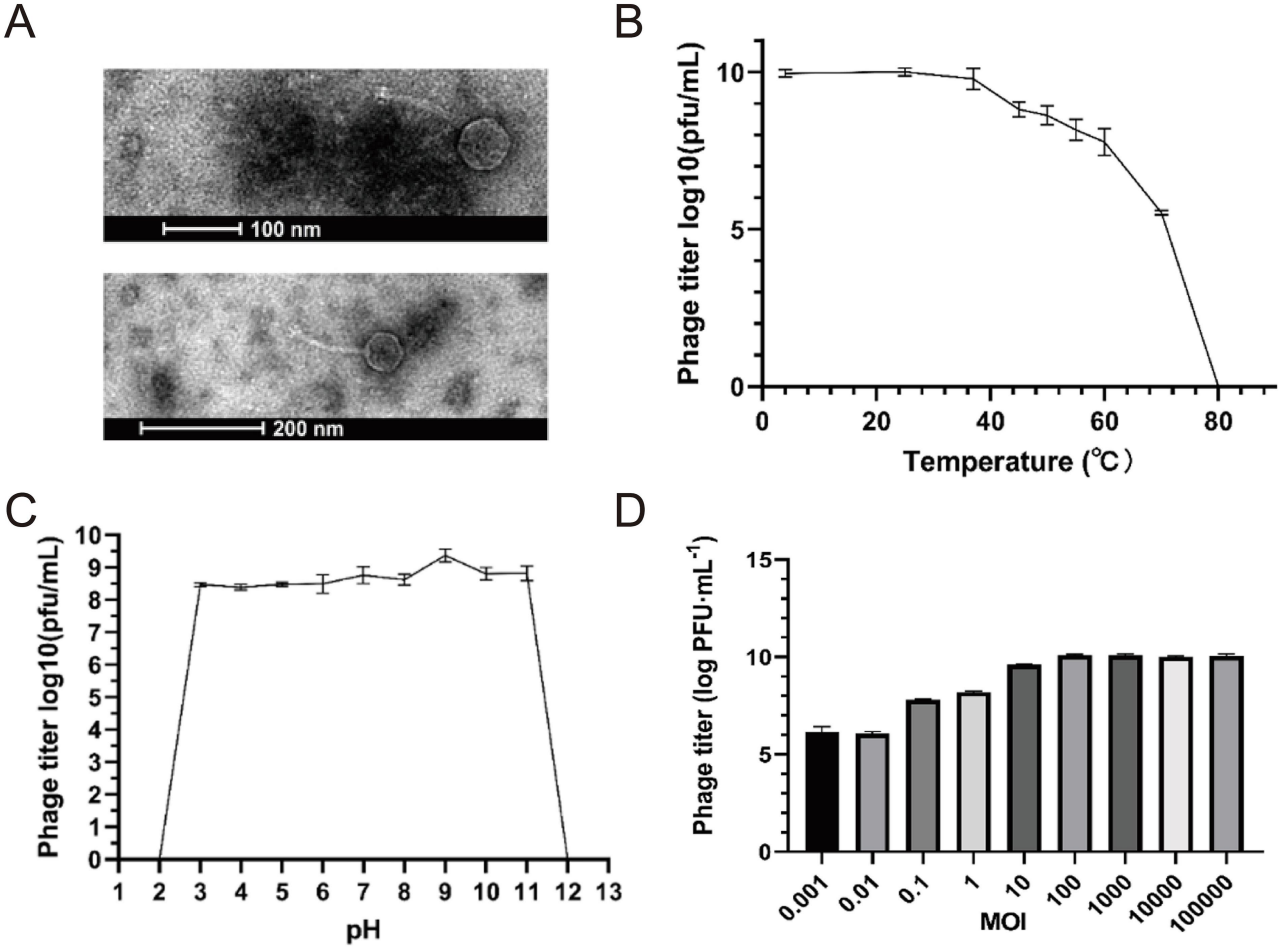
Basic characteristics of Escherichia phage vB_Eco_K1B4. **(A)** Negative staining results showed that the head diameter of vB_Eco_K1B4 was 54.13 ± 0.15 nm, presenting a regular polyhedral shape, and it had a non-contractile tail of length 142.10 ± 0.43 nm, classifying it as a member of the long-tailed phages. **(B)** Thermal stability curve of vB_Eco_K1B4. The x-axis denotes the temperature, and the y-axis shows the phage titer following a 1-h incubation period in a water bath at each specified temperature. **(C)** pH Stability Curve of vB_Eco_K1B4. The x-axis denotes the pH values, and the y-axis shows the phage titer following a 1-h incubation period at 37 °C at each specified pH value. **(D)** MOI of vB_Eco_K1B4 *Escherichia coli* K1B4, comparing the number of phages produced at different initial ratios of phage to bacteria.

The purified phages were enriched by the double-layer agar plate method (Liu et al., 2019; Li et al., 2020) as follows: Phage vB_Eco_K1B4 with the potency of 1 × 10^8^ PFU/mL was used for morphological observation. Restaining was performed using 2% phosphotungstic acid, followed by observation of the morphology of the phage using transmission electron microscopy. Negative staining electron microscopy revealed that phage vB_Eco_K1B4 has an icosahedral head (54.13 ± 0.15 nm in diameter) and a long, non-contractile tail (142.10 ± 0.43 nm) (Figure 1A), which is morphologically typical of the siphovirus morphotype. However, based on comprehensive genomic and phylogenetic evidence, it is classified under the genus Kagunavirus (subfamily Guernseyvirinae).

Subsequently, we analyzed the biological characteristics of the bacteriophage. The thermal stability curve of vB_Eco_K1B4 showed that the phage was best active at 25 °C. The survival rate was 55.24% after incubating 1 h of water bath at 70 °C, which means bacteriophage gradually decreased with the temperature increase. Under the 80 °C condition of a water bath for 1 h, the vB_Eco_K1B4 was completely inactivated. We found that the phage still had high activity at 4 °C so that vB_Eco_K1B4 could be stored at 4 °C for a short period (Figure 1B). This indicates that bacteriophage B4 not only can withstand high temperatures but also has high adaptability to low temperatures. The vB_Eco_K1B4 survived well from pH 3 to 11 and had the highest activity at pH 9. We have wholly inactivated the bacteriophage at highly acidic and alkaline conditions (pH 2 or 12) (Figure 1C). This proves that the bacteriophage has a wide pH range. VB_Eco_K1B4 is a bacteriophage isolated from the pig intestine. The average pH value in the pig stomach is 4.8, the average pH value in the small intestine is 6.7, and the average pH value in the large intestine is around 7.5 (Barmpatsalou et al., 2021). Therefore, we speculate that the wide pH range of vB_Eco_K1B4 may be related to its adaptation to the pH of the pig digestive system.

The appropriate multiplicity of infection (MOI) value is one of the critical factors for high infection efficiency. The phage titer was determined by mixing bacteriophage vB_Eco_K1B4 and *E. coli* B4 and comparing the number of phages produced at different MOI. The maximum phage titer was 10^9^ PFU/mL at MOI of 100 (Figure 1D). Under this MOI condition, the ability of bacteriophage vB_Eco_K1B4 to infect the host is strong.

### 3.2 Genome sequence of host K1B4 and bacteriophage vB_Eco_K1B4

We sequenced the whole genome of strain TW11 to understand the host versus phage genome. The chromosome size of *Escherichia coli* K1B4 was 4,594,027 bp (51% GC), containing 4,410 predictive coding sequences (CDSs), 22 rRNAs, and 86 tRNAs.

In addition, the chromosome size of the K1B4_plasmid1 is 81,614 bp (52% GC) and contains 93 predicted coding sequences (CDSs). The chromosome size of the K1B4_plasmid2 was bp (49% GC) and contained 54 predicted coding sequences (CDSs).

**Figure 2:**
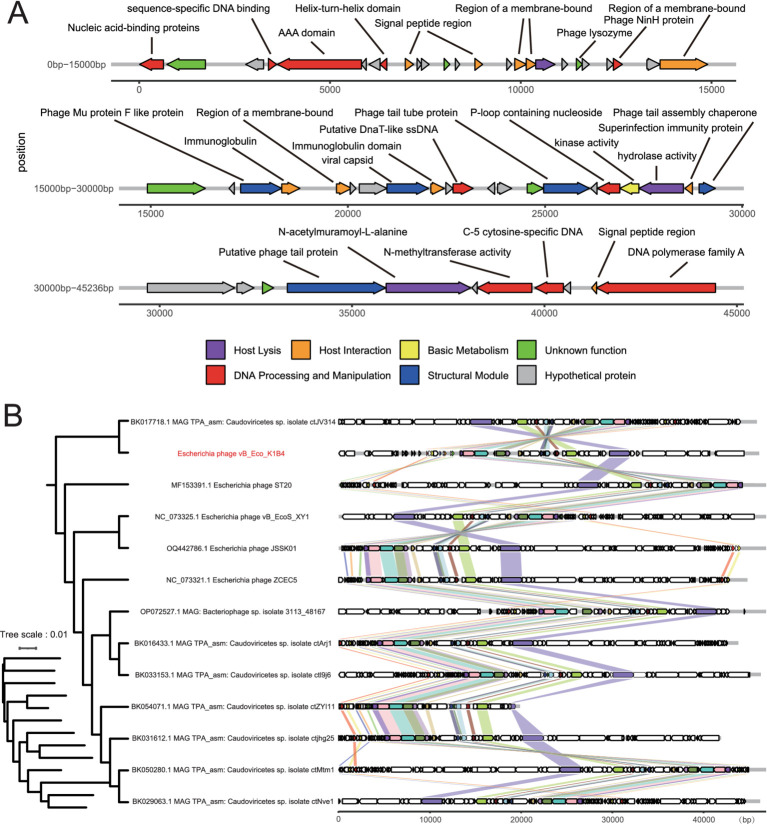
Genome features of phage vB_Eco_K1B4. **(A)** Genome structure of vB_Eco_K1B4. The genome structure of phage XX features all predicted open reading frames (ORFs), which have been classified into six categories based on their predicted functions. These ORFs are color-coded and represented with arrows indicating their transcriptional orientation within the genome. **(B)** Phylogenetic tree and comparative genomics of phage vB_Eco_K1B4. The phylogenetic tree of phage XX, along with comparative genomics analyses of its genome relative to those of other phages, has been constructed using OrthoFinder. In this tree, each arrow signifies an individual ORF, facilitating the examination of evolutionary relationships and genomic similarities among phages, including phage vB_Eco_K1B4.

The phage vB_Eco_K1B4 is a cyclic phage with a genome length of 44,581 bp and 50.64% of (G + C); 24.51, 24.85, 25.45, and 25.19% of A, T, G, and C, respectively. We compared the vB_Eco_K1B4 with the phage sequences from NCBI and found that the nucleotide similarity was less than 95%. According to the International Committee on Taxonomy of Viruses (ICTV) classification, vB_Eco_K1B4 belongs to a novel strain of *E. coli* phage that degrades the K1B4 capsule. According to gene function prediction, this bacteriophage has 57 open reading frames (ORFs) and 0 tRNA genes. The genome did not contain antibiotic resistance genes or virulence factors. Among the 57 predicted ORFs, only part of the ORFs were potentially annotated for their functions (Supplementary Table S1). Protein function prediction of phage vB_Eco_K1B4, including energy utilization module, tail structure, head structure, cleavage module, DNA replication, recombination, modification module, and DNA packaging module; the remaining ORFs were proteins of unknown functions. Subsequently, we mapped the whole genome of vB_Eco_K1B4. Since nearly this bacteriophage has low homology with other phages, it is evident that the heterogeneity of the genes of this bacteriophage is high, and further studies and functional validation are needed (Figure 2A).

To understand the evolutionary relationships between phage vB_Eco_K1B4 and other phages, relatively conserved and evolutionarily significant genes were selected for phylogenetic tree analysis (Figure 2B and Supplementary Figure S5). The collinearity analysis of bacteriophages in the phylogenetic tree was carried out. The results of the analysis show that phage vB_Eco_K1B4 was closely related to phage ST20 (isolated in 2017, MF153391.1), and phage vB_EcoS_XY1 (isolated in 2020, NC_073325.1). The ST20 and vB_EcoS_XY1 all belonged to the subfamily *Guernseyvirinae* and *genus Kagunavirus*. Based on the evolutionary relationships, vB_Eco_K1B4 should belong to the *Kagunavirus virus.* vB_Eco_K1B4 was also taxonomically annotated by vConTACT2 software. Consistent with the transmission electron microscopy observations, it belongs to a strain of the *Siphoviridae* family (long-tailed) phage. Also, in agreement with the results of phylogenetic tree analysis, vB_Eco_K1B4 belongs to a strain of the *genus Kagunavirus.* The results of collinearity analysis showed that the prediction characteristics of some vB_Eco_K1B4 were highly similar to those of some genes on other phages in the phylogenetic tree. Three of these genes can be annotated functional, as shown in the gene structure diagram (Figure 2A), vB_Eco_K1B4_023 can be classified as DNA combination, identification, replication, recombination and modification, vB_Eco_K1B4_025 and vB_Eco_K1B4_028 are related to the synthesis of phage structure.

Besides, this study annotated 48 antimicrobial resistance genes in *Klebsiella pneumoniae* strain K1B4 using the CARD database (Supplementary Table S3). The genes primarily conferred resistance to *β*-lactams (e.g., penicillin, cephalosporin), macrolides (e.g., erythromycin), fluoroquinolones (e.g., ciprofloxacin), and peptide antibiotics (e.g., polymyxin B). Dominant mechanisms included efflux pumps (85% of genes, predominantly RND and MFS types) and antibiotic target modifications (15%). Notably, acr, mdt, and tolC gene families exhibited cross-resistance to 5–12 antibiotic classes, while arnT and pmrF mediated critical polymyxin resistance. Annotation quality was robust, with 90% of genes showing Perfect/Strict matches and >97% sequence identity.

### 3.3 Infection profile and transcriptomic dynamics of bacteriophage vB_Eco_K1B4

**Figure 3:**
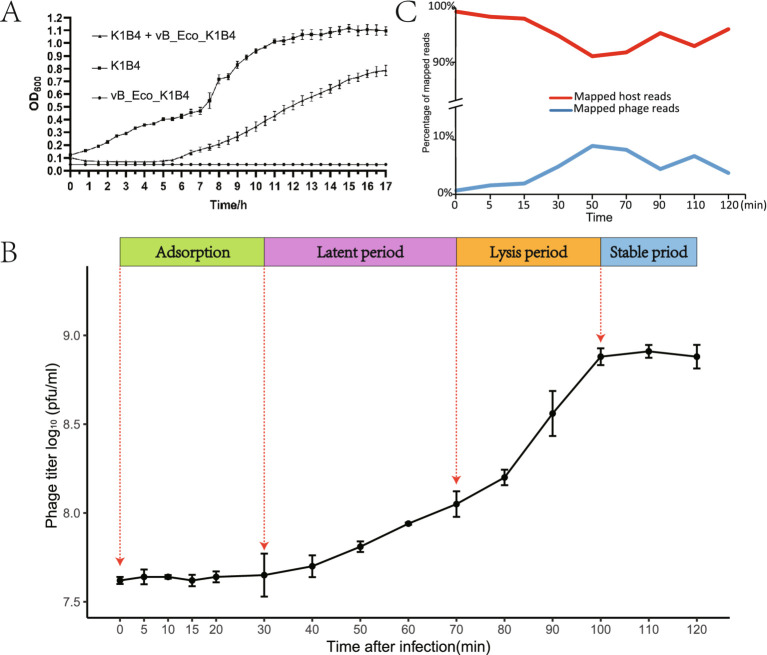
The lysis cycle of phage vB_Eco_K1B4 on *Escherichia coli* K1B4. **(A)** Viability of *Escherichia coli* K1B4 following infection by phage vB_Eco_K1B4. The viability of *Escherichia coli* K1B4 following infection by phage vB_Eco_K1B4 was assessed through the measurement of optical density (OD) at 600 nm for samples collected at specified time points post-infection. This approach provides insight into the survival and growth dynamics of the bacterial host in response to phage infection. **(B)** One-step growth curve of phage vB_Eco_K1B4. The one-step growth curve of phage vB_Eco_K1B4 encompasses a timeframe from 0 to 120 min and delineates four distinct phases: adsorption, latent, lysis, and stationary periods. Red dashed arrows mark the starting time points of each phase, facilitating the characterization of key stages in the phage life cycle. **(C)** Percentage of RNA reads for host (red) and Phage vB_Eco_K1B4 (Blue) genomes at different time points post-infection. Statistical analysis was conducted to determine the percentage of RNA reads for the genomes of host (red) and phage vB_Eco_K1B4 (blue) at different time points following infection by phage X. This analysis provides valuable information on the transcriptional dynamics of both the host and the phage during the infection process.

To study the infection pattern of the phage vB_Eco_K1B4, we analyzed its *in vitro* lysis curve and one-step growth curve (Figures 3A,B). The phage vB_Eco_K1B4 and the host bacterium B4 were incubated with the optimal MOI mixture for 17 h. The vB_Eco_K1B4 completely inhibited the growth of the host bacterium B4 in the first 4.5 h, while the host bacterium without phage maintained continuous growth and entered the plateau period after about 14.5 h (Figure 3A). This indicates that bacteriophage vB_Eco_K1B4 has a strong killing ability against K1B4 and can effectively inhibit the growth of K1B4 (Saunier et al., 2024).

The one-step growth curve of phage vB_Eco_K1B4 was shown in Figure 3B, there was no significant change in the potency of the phage in the first 30 min, and the potency of the phage increased sharply in 30–100 min and then stabilized. Therefore, the latent period of phage vB_Eco_K1B4 is 30 min and the outbreak period is 70 min.

The highest potency of phage can reach 8.1 × 10^8^ PFU/mL. From the amount of outbreak = the number of phages at the end/the number of bacteria at the beginning, it is known that the outbreak amount of phage vB_Eco_K1B4 is 72.27 (Figure 3B).

Based on the infection cycle of the phage vB_Eco_K1B4, we selected 8 time points for RNA-seq analysis, with 0 min as the control and three replicates. We found that the phage transcript was gradually replacing the host transcript from 0 min, and the phage transcript increased from 0.7% at 0 min to 8.89% at 50 min, reaching the peak.

The proportion of host transcripts decreased from 99.3% at 0 min to 91.1% at 50 min (Figure 3C). This suggests that bacteriophage B4 infection may effectively redirect the host’s transcriptional resources towards phage replication, as evidenced by the overall downregulation of host transcripts.

### 3.4 Temporal expression patterns of bacteriophage vB_Eco_K1B4

**Figure 4:**
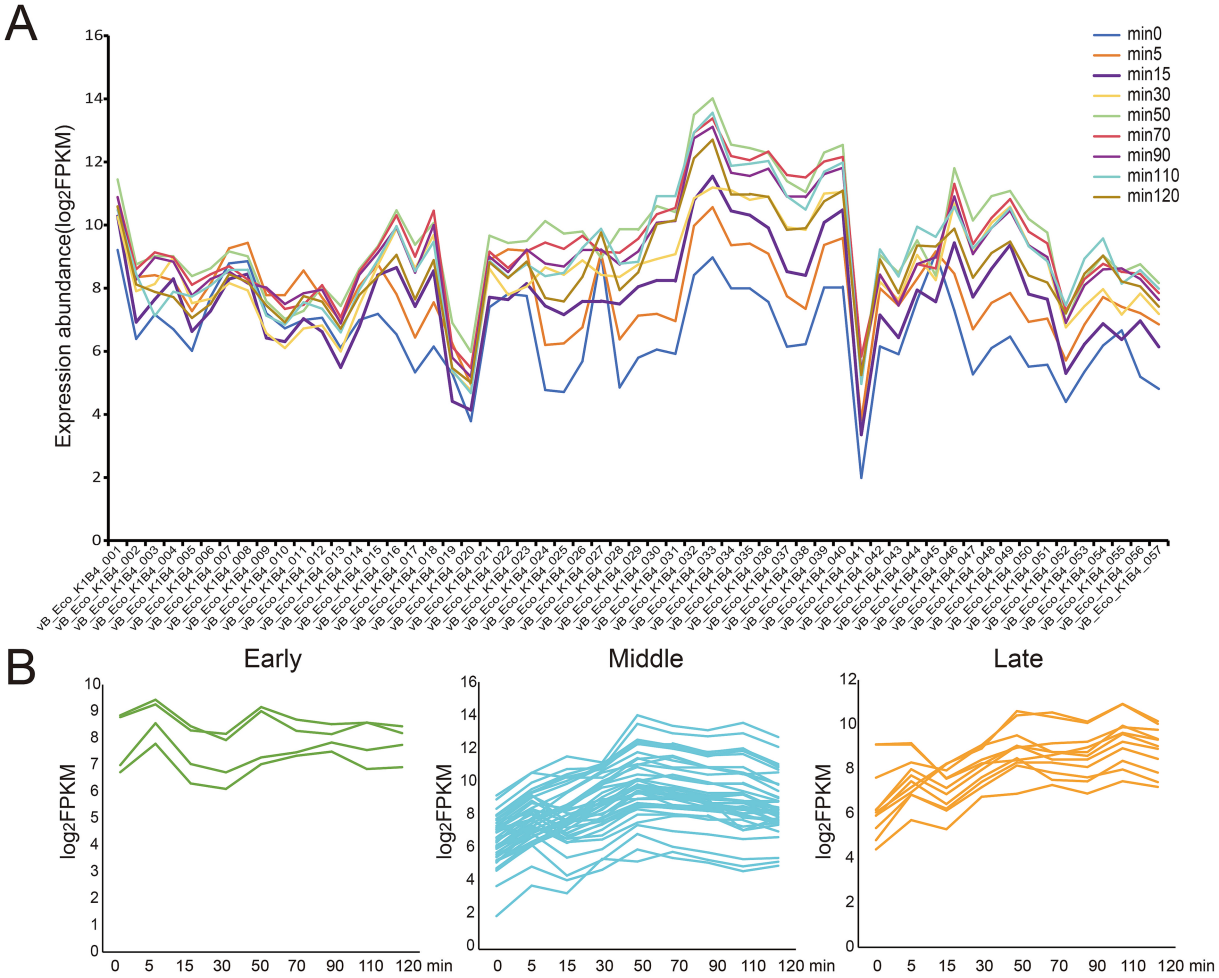
Transcriptional profile of phage vB_Eco_K1B4. **(A)** Expression patterns of phage vB_Eco_K1B4. Based on the expression abundance of phage vB_Eco_K1B4 genes, we roughly categorized them into three expression patterns. Genes that reach their expression peak within the first 30 min are designated as early genes. Those that peak between 30 and 100 min post-infection are termed middle genes. Finally, genes that achieve their peak expression after 100 min are classified as late genes. **(B)** Expression profiles over time. Graphs displaying the expression profiles of individual genes within each expression category are shown as a function of time following infection. These descriptions provide a clear outline of the temporal dynamics of gene expression in phage vB_Eco_K1B4 during the course of its infection cycle.

Using the one-step growth curve (Figure 3B) of the phage vB_Eco_K1B4 as a reference, we divided the 8 time points used for RNA-seq into 3 periods. Among them, 5 min, 15 min and 30 min are classified as early stage, 50 min, 70 min and 90 min are classified as middle stage, and 110 min and 120 min are classified as late stage. Then, according to the order of transcriptional expression peaks (Figure 4A), we classified 57 genes in the phage vB_Eco_K1B4 into the above three periods, namely 4 genes in the early stage (Figure 4B, green), 42 genes in the middle phase (Figure 4B, blue), and 11 genes in the late stage (Figure 4B, yellow). Only vB_Eco_K1B4_008 in the early genes are annotated to function, which is the Helix-turn-helix domain (Kelley and Yanofsky, 1985), a DNA-binding domain (literature, first discovered in lambda phages). Most of the metaphase genes are related to phage replication and synthesis, such as specific DNA binding (vB_Eco_K1B4_004), AAA domain (vB_Eco_K1B4_005), and phage.

NinH protein (vB_Eco_K1B4_023). These genes mainly play an important role in DNA binding, recognition, replication, recombination, and modification. Similarly, after the phage regulates the host’s transcriptional resources to serve itself, it will begin to use the host to synthesize new phage structural proteins, such as Phage Mu protein F-like protein (vB_Eco_K1B4_028), viral capsid (vB_Eco_K1B4_033), phage tail protein (vB_Eco_K1B4_050), etc. The expression of these genes provides an important source of structural components for phage replication. At the same time, some genes associated with cell lysis, such as bacteriophage lysozyme (vB_Eco_K1B4_018), are gradually being expressed, suggesting that some of the infected hosts may have been lysed or are about to be lysed. Not only that, but vB_Eco_K1B4_051 also reached its peak expression during this period, and this gene is involved in the regulation of Nacetylwallyl-L-alanine amide activity, which is also involved in cell lysis (related literature is in the folder). This suggests that during this period, the phage may be using the host’s transcriptional resources to perform a large amount of self-replication, and after completion, the host is lysed and released extracellularly for the next round of infection. Finally, the functions of advanced genes are mainly focused on cell lysis and DNA replication, for example, vB_Eco_K1B4_045 expression synthesized a superinfection immunity protein that is involved in cell lysis (Lu and Henning, 1989; He et al., 2024). At the same time, vB_Eco_K1B4_053, vB_Eco_K1B4_054, and vB_Eco_K1B4_057 are involved in the synthesis or regulation of N-methyltransferase activity, C-5 cytosine-specific DNA methylase, and DNA polymerase family A (Mollner and Braun, 1984).

### 3.5 Host response to infection by bacteriophage vB_Eco_K1B4

**Figure 5:**
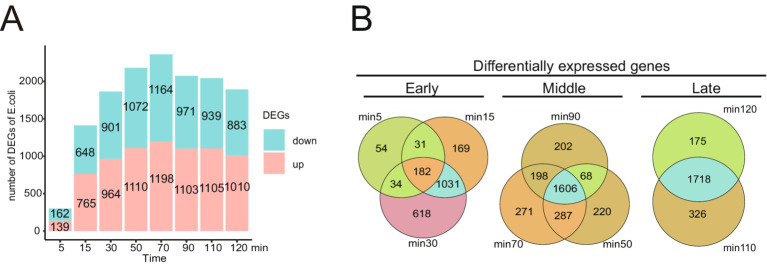
Functional analysis and differentially expressed genes between phage vB_Eco_K1B4 and *Escherichia coli* K1B4. **(A)** Number and distribution of DEGs across different infection stages. This section illustrates the number and distribution of differentially expressed genes (DEGs) across various stages of infection. It provides insight into the changes in gene expression dynamics during the progression of the infection process. **(B)** Venn diagram showing the intersection of DEGs across different stages. Venn diagram is used to display the intersection of DEGs across different infection stages, highlighting the shared and unique DEGs among the sampled time points. This analysis helps elucidate the temporal specificity and commonality of gene expression changes during the infection lifecycle.

Differentially expressed genes (DEGs) analysis. In order to investigate the response of the host to bacteriophage vB_Eco_K1B4 infection, we conducted differential analysis of host genes and counted the differentially expressed genes at 8 selected time points (Figure 5A). Statistics show that, except for the 5th minute, the number of differentially expressed genes is more upregulated than downregulated. In addition, the total number of differentially expressed genes gradually increased and reached its peak at 70 min. It is worth noting that the three periods with the highest number of differentially expressed genes (50 min, 70 min, and 90 min) are the middle period of the three periods we divided based on the one-step growth curve (Figure 2B), and according to the Venn diagram (Figure 5B), most of the differentially expressed genes are common to these three time points during the middle period, reaching 1,606 DGEs.

Similarly, this phenomenon also occurred in the two late stages (110 min and 120 min) (Figure 5B), where most of the differentially expressed genes were shared between these two stages, reaching 1718 DEGs, while specific genes accounted for a minority. This may be because the host had already reached a stable period of phage infection during this period, and the interval between these two time points was relatively short. It is worth noting that there were only 182 differentially expressed genes in the early three stages (5-min, 15 min, and 30 min), but the number of differentially expressed genes in the 15 min and 30 min stages reached 1,213. This phenomenon may be caused by a large number of bacteriophages still in the early stage of infection at the 5th minute, and the bacteriophages have not yet seized the host’s transcription resources in large quantities. Therefore, during this time point, most of the host genes are still in a normal expression state.

Functional analysis of host DEGs. Subsequently, to gain a more detailed understanding of the functions of differentially expressed genes, we performed COG functional annotation on the host and its plasmid, as well as the bacteriophage vB_Eco_K1B4 (Supplementary Figure S6 and Supplementary Table S2). We focused on the COG functional annotation of shared genes at different time points in each period. The results showed that in the early host genes, the shared upregulated genes at three time points mainly focused on energy production and transformation, amino acid transport, and metabolism, while the downregulated genes mainly focused on carbohydrate transport and metabolism. However, considering that the phage had not yet extensively infected the host at 5 min, we separately counted the shared genes at 15 min and 30 min. The COG annotation results showed that the up-regulated genes were still concentrated in energy production and transformation, amino acid transport, and metabolism at these two time points, but the functions of downregulated genes were mainly focused on translation, ribosome structure and biogenesis, transcription, and cell wall/membrane/envelope biogenesis. In the mid-term host genes, shared upregulated genes are not only concentrated in energy production and conversion, amino acid transport and metabolism, but also in carbohydrate transport and metabolism, inorganic ion transport and metabolism. The shared downregulated genes are similar to the 15 min and 30 min shared genes, focusing on translation, ribosome structure and biogenesis, transcription, and cell wall/membrane/envelope biogenesis. In the late stage (110 min & 120 min), the main functions of upregulated and downregulated genes are similar to those of differentially expressed genes in the mid-stage.

In addition, according to GO analysis, we found that at all time points, pathways related to energy metabolism, such as energy export from organic compound oxidation, precursor metabolites, and energy production, were upregulated. The downregulated pathways were the assembly of membrane-less organelles, peptide biosynthesis processes, etc. (Figure 1), which is similar to the results of COG annotation, that is, the upregulated parts are mostly related to energy, while the downregulated parts can indirectly confirm that host related transcription resources have been replaced by bacteriophages. For detailed data on other pathways, please refer to Figure 1.

#### 3.5.1 Analysis of the co-expression network of vB_Eco_K1B4-K1B4

**Figure 6:**
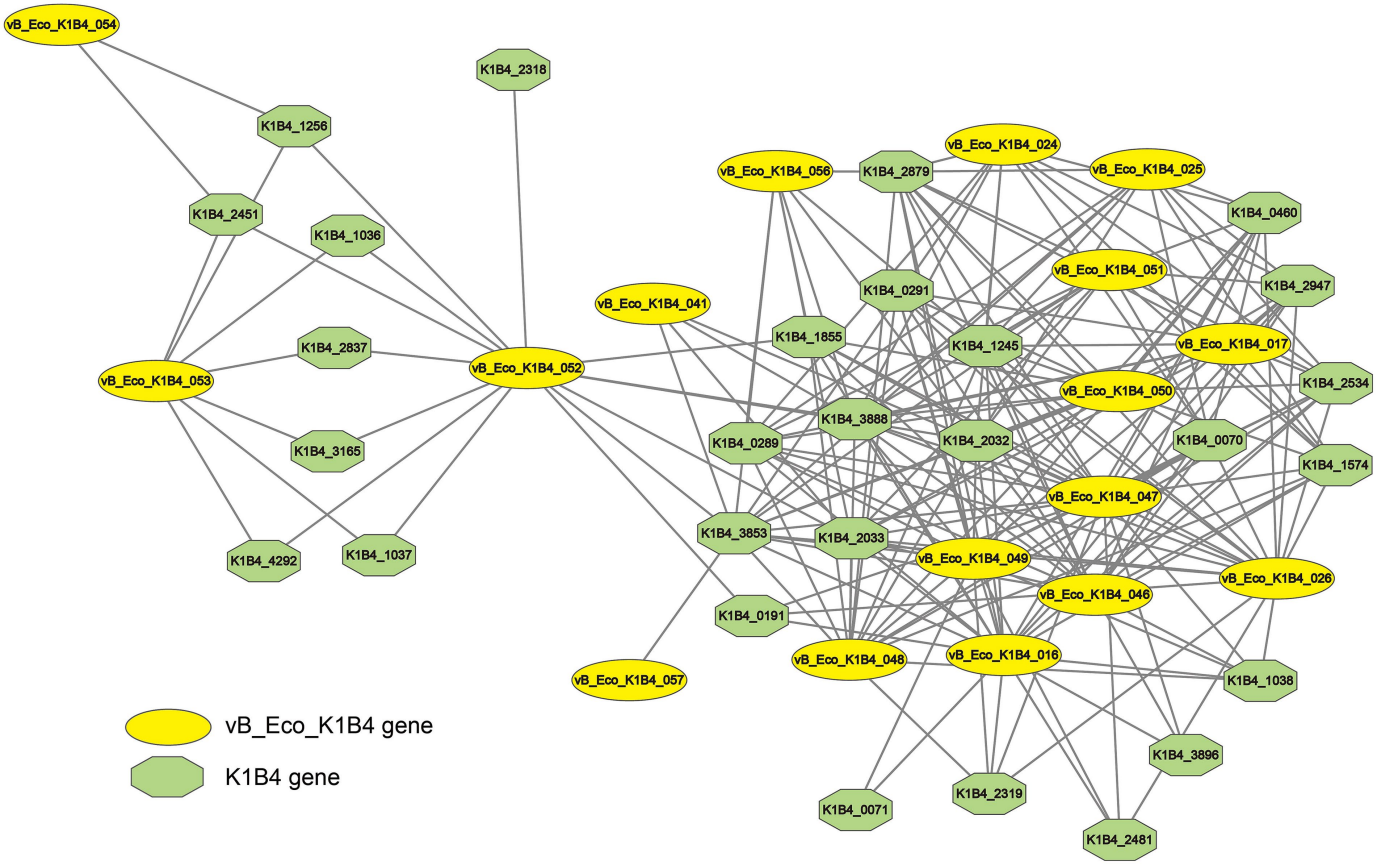
Co-expression network between phage vB_Eco_K1B4 and its host. The interactions between phage vB_Eco_K1B4 genes (represented in yellow) and host genes (represented in green).

To investigate the interaction between bacteriophages and host gene expression, we constructed an interaction network (Figure 6). Through co-expression network analysis, we identified 17 bacteriophage vB_Eco_K1B4 genes and 28 host genes. 6 genes can be annotated by eggnog in the node of vB_Eco_K1B4, and the annotated phage genes can be roughly divided into two categories. The main functions of the first type of genes are related to the structure of bacteriophages, including bacteriophage tail protein (vB_Eco_K1B4_050), bacteriophage tail assembly partner protein family (vB_Eco_K1B4_046) (A conserved spiral structure for high; Eco_K1B4_025). In addition, all host genes within the co-expression network can be annotated, and their specific functions can be found in the Supplementary Table.

#### 3.5.2 Response of host antiphage mechanisms after vB_Eco_K1B4 infection

After analyzing the function of the differentially expressed genes, we found an interesting phenomenon in which some of the host K1B4 genes used to defend against phage infection showed a down-regulated trend. The representative genes among these downregulated defense genes are HigB-HigA, which are a group of toxin-antitoxin systems (TA systems). A primary physiological role of toxin-antitoxin systems is phage inhibition (Pell et al., 2013; Jadhav et al., 2020). Among them, higB-higA is a representative pair of type II toxin-antitoxin systems, and it has been reported that higB can selectively cleave ribosome-bound mRNA to regulate protein production when activated (the mechanism by which HigB toxin cleavages endonucleases), which is a great threat to phages when they compete for host transcription resources (Schureck et al., 2016; Song and Wood, 2020). HigA, on the other hand, can inhibit the toxicity of higB by binding to higB (resolution structure of *E. coli* HigBA). We analyzed the expression of higB-higA after phage infection of the host, and found that the expression of higB showed a down-regulated trend from 15 min, while the expression of higA showed a down-regulated trend from 30 min (Supplementary Figure S4). The observed concurrent downregulation of both higB (toxin) and higA (antitoxin) genes following phage infection is a notable finding. While the precise mechanism remains unclear, this pattern is consistent with a scenario where vB_Eco_K1B4 infection may indirectly or directly interfere with the host’s TA systems, potentially as a means to circumvent this defense barrier. However, it is important to note that this remains a correlation observed via transcriptomics; future studies involving genetic knockout of the higBAlocus will be essential to functionally validate its role in the defense against vB_Eco_K1B4 and to establish any causal relationship.

### 3.6 Verification of DEGs by RT-qPCR

**Figure 7:**
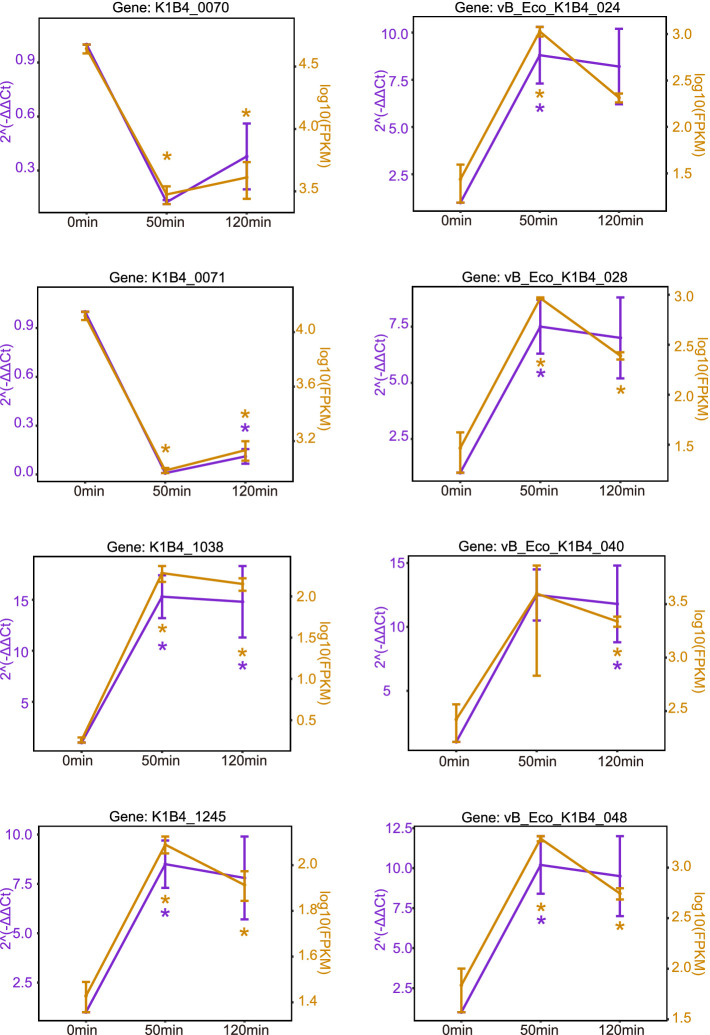
Validation of RNA-seq results by RT-qPCR. The expression levels of selected genes were measured by RT-qPCR in three independent biological replicates. The relative expression (RT-qPCR) was calculated using the 2^–ΔΔCT^ method and normalized to the endogenous gene vB_Eco_K1B4. Data are presented as the mean ± SD (*n* = 3). Statistical significance was determined by an unpaired Student’s *t*-test, comparing each time point to the 0 min control (*p < 0.05; **p < 0.01). For comparison, the corresponding log10 (FPKM+1) values from the RNA-seq analysis are shown alongside.

To ascertain the reliability of the RNA-sequencing (RNA-seq) data, we selected ten K1B4 genes and six phage genes for validation via RT-qPCR. The analysis revealed that the trends in the expression patterns observed in the RNA-seq data were consistent with those obtained from RT-qPCR (Figure 7). This concordance substantiates the reliability of the RNA-seq results.

## 4 Discussion

In animal husbandry, the presence of pathogenic microorganisms often brings huge losses. In the past, people usually relied on the use of antibiotics to prevent pathogenic microorganisms that cause huge losses in livestock production, although this method can inhibit their growth, but also cause contamination of agricultural products, not to mention the increase in microbial pathogen resistance, the focus of solving the problem of bacterial resistance is gradually shifting to phage therapy (Chegini et al., 2020; Chegini et al., 2021; Ibrahim et al., 2021). However, phage therapy should have some good properties, such as they should preferably be lytic, kill the bacterial host efficiently, and be fully characterized to exclude side effects (Strathdee et al., 2023; Wen et al., 2023). This requires a more accurate and comprehensive analysis of the interaction mechanism between a variety of bacteriophages and their specific hosts. Therefore, in order to gain a deeper understanding of the interaction between bacteriophages and pathogenic microorganisms in the pig gut, we isolated a novel bacteriophage vB_Eco_K1B4 from the pig gut and studied its biological characteristics and temporal transcriptomic interaction with the host *E. coli* K1B4.

Biological characterization shows that bacteriophages vB_Eco_K1B4 viruses belonging to the family of long tailed Bacteriophages, which have a high tolerance to high and low temperatures. Not only that, but vB_Eco_K1B4 also has a wide pH range (4.8 to 7.5), so we speculate that the reason why vB_Eco_K1B4 has a wide pH range may be related to the vB_Eco_K1B4 adaptation to the pH in the pig digestive system. Subsequently, we demonstrated that the vB_Eco_K1B4 could effectively inhibit the growth of host K1B4 under the condition of optimal MOI (MOI = 100). The time required for different phages to pass the incubation period and release the next generation of phages varies, The incubation period of phage ST20, which has a phylogenetic similar relation to vB_Eco_K1B4, is about 38 min at 37 °C (Liu et al., 2019), while the incubation period of vB_Eco_K1B4 is roughly 30 min. In addition, both ST20 and vB_Eco_K1B4 can be stable at 4 °C. Therefore based on the above characteristics, vB_Eco_K1B4 has great potential for application.

Similar to other bacteriophages (Yang et al., 2019; Li et al., 2020; Li et al., 2022), vB_Eco_K1B4 begins to compete for host transcriptional resources after infecting the host. However, unlike other reported bacteriophages, vB_Eco_K1B4 only needs to encroach on a small portion of the host’s transcriptional resources to complete its own replication. In addition, the results of differentially expressed genes analysis showed that phage vB_Eco_K1B4 can regulate the expression of host genes in a very precise manner (Gong et al., 2024). As shown in the COG annotation, the up-regulated differentially expressed genes of host K1B4 are concentrated in energy metabolism and substance transport throughout the process, while the down-regulated genes are concentrated in transcription, translation, and synthesis of some host structural components. Similarly, GO enrichment analysis of host differentially expressed genes showed that the up-regulated pathways throughout the entire process were related to pathways such as energy metabolism, while the down-regulated pathways involved the synthesis of the host’s components. At the same time, the gene functions of bacteriophage vB_Eco_K1B4 are mainly focused on the synthesis of structural components, DNA modification, and replication. In addition, the co-expression network also showed that the node genes of the vB_Eco_K1B4 were also related to the synthesis of structural components and DNA modification, replication, etc., which is consistent with the above results. All of the above evidence indicates that bacteriophage vB_Eco_K1B4 can regulate the expression of host genes in a very precise manner, similar to some reported bacteriophages (Li et al., 2020).

It is worth noting that we found differential expression of anti-phage genes in some hosts after infection of host K1B4 by bacteriophage vB_Eco_K1B4, with HigB HigA being a typical example. According to existing reports (Fivian-Hughes and Davis, 2010; Schureck et al., 2016; Qi et al., 2021), HigB-HigA is a type II TA system (toxinantitoxin system) in which HigB can selectively cleave mRNA and interfere with the transcriptional requirements of phages invading the host and then infect other bacteria. Similarly, phages have evolved countermeasures to counteract the host’s antiviral system (Samson et al., 2013). In our analysis, we found that the expression of HigB has been consistently down-regulated, indicating that vB_Eco_K1B4 may have inhibited the expression of HigB through some mechanism, which greatly reduces the defense ability of K1B4 against vB_Eco_K1B4 infection. However, the specific mechanism of this action still needs to be studied.

There are inevitably some shortcomings in this study. Firstly, we still need more data to analyze these mechanism as to how vB_Eco_K1B4 regulates the expression of host genes specifically. Furthermore, further experiments and analysis are needed to demonstrate how vB_Eco_K1B4 avoids hunting by host defense mechanism. Finally, this study only examines the transcriptome of vB_Eco_K1B4 interactions with K1B4, so there is still a lot to be studied, such as proteomic and metabolomic interaction.

## 5 Conclusion

In summary, we isolated a novel bacteriophage vB_Eco_K1B4 and studied its biological characteristics, as well as the transcriptional interaction between the phage and the host *E. coli* K1B4. We found that vB_Eco_K1B4 can effectively utilize the host’s transcription resources to complete self-replication, while also effectively inhibiting the expression of some host genes. Moreover, vB_Eco_K1B4 can effectively inhibit some host defense mechanism. This study enhances our understanding of the interaction between bacteriophages and hosts in pig intestines, and further research to verify these conclusions will be of great significance.

## Data Availability

The raw sequence data reported in this paper have been deposited in the Genome Sequence Archive (Chen et al., 2021) at the National Genomics Data Center (CNCB-NGDC Members and Partners, 2022, 2024), China National Center for Bioinformation/Beijing Institute of Genomics, Chinese Academy of Sciences under accession number PRJCA028359, and in the NCBI GenBank under BioProject accession number PRJNA939978. These datasets are publicly accessible at https://ngdc.cncb.ac.cn/gsa and https://www.ncbi.nlm.nih.gov/bioproject/PRJNA939978, respectively.

## Supplementary material

SUPPLEMENTARY FIGURE S1 GO enrichment analysis of host differentially expressed genes at different time points (BP).

SUPPLEMENTARY FIGURE S2 GO enrichment analysis of host differentially expressed genes at different time points (CC).

SUPPLEMENTARY FIGURE S3 GO Enrichment analysis of host differentially expressed genes at different time points (MF).

SUPPLEMENTARY FIGURE S4 Expression levels of the host's toxin-antitoxin system HigB-HigA following phage vB_Eco_K1B4 infection.

SUPPLEMENTARY TABLE S1 Basic information of the co-expression network between host and phage.

SUPPLEMENTARY TABLE S2 Genome annotation of phage vB_Eco_K1B4.

SUPPLEMENTARY TABLE S3 Antimicrobial resistance genes identified in Escherichia coli K1B4 by CARD analysis.
