# Supplementary figures and images for: Temporal transcriptomics uncover dynamic interactions between pathogenic Escherichia coli and phage vB_Eco_K1B4
Source: Front Microbiol. 2025 Nov 11;16:1668341. doi: 10.3389/fmicb.2025.1668341 (PMC12645636; doi:10.3389/fmicb.2025.1668341)

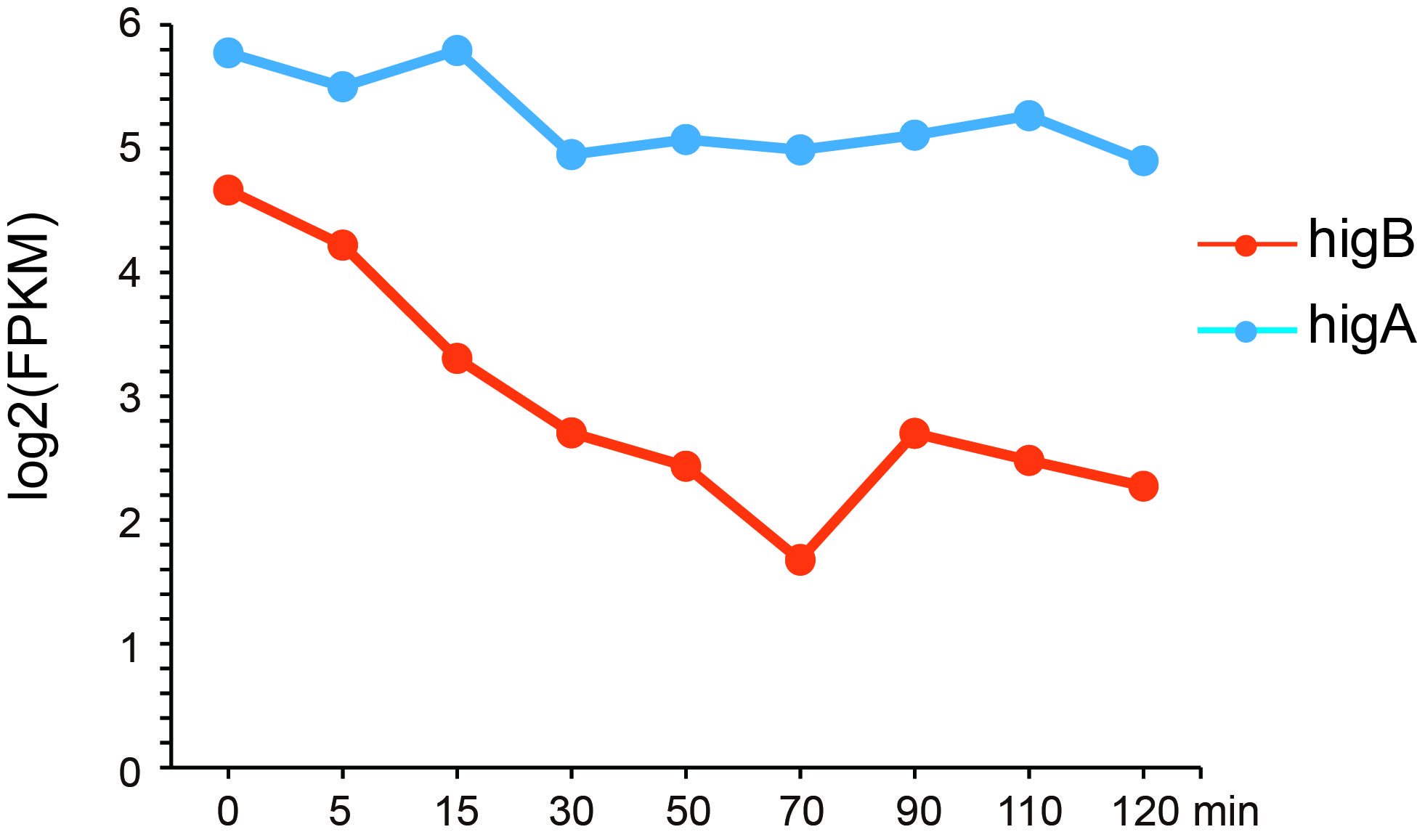

Supplement: SUPPLEMENTARY FIGURE S4 — Expression levels of the host's toxin-antitoxin system HigB-HigA following phage vB_Eco_K1B4 infection. [file Image_4.jpeg]

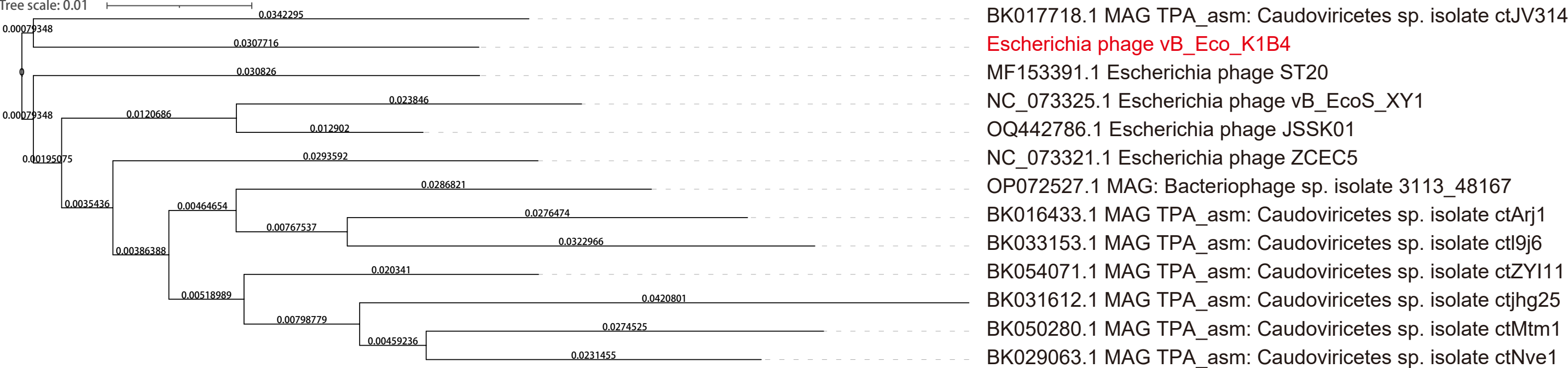

Supplement: Supplementary file 8 [file Image_5.pdf]

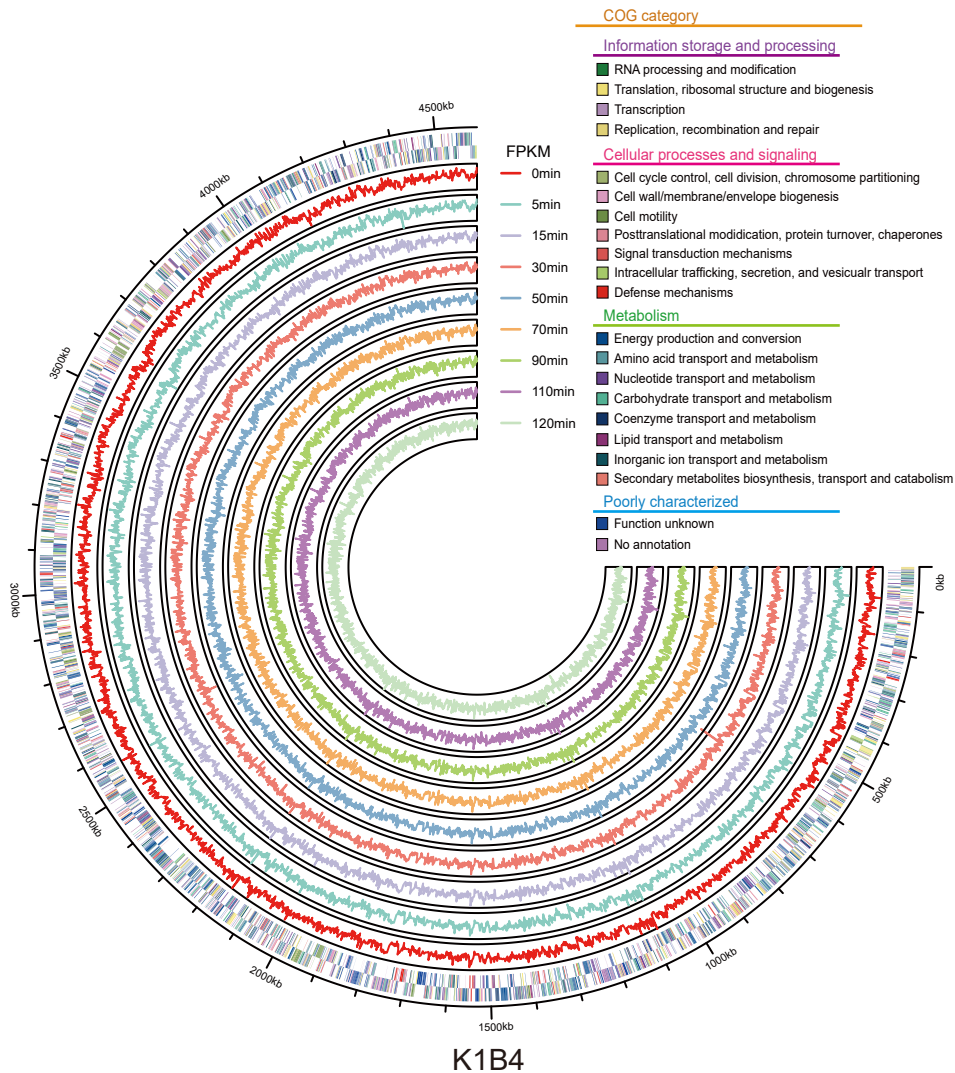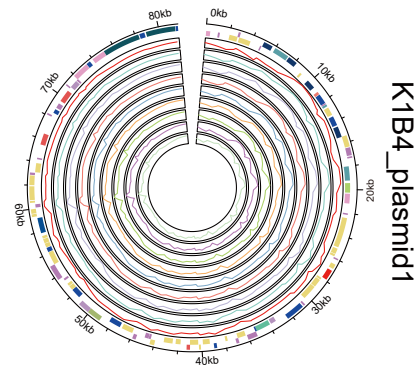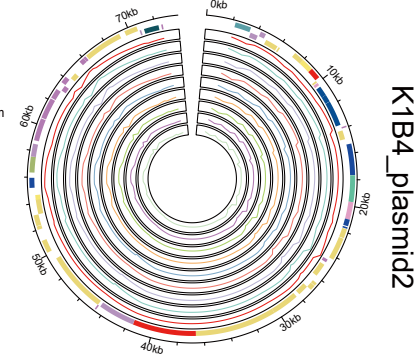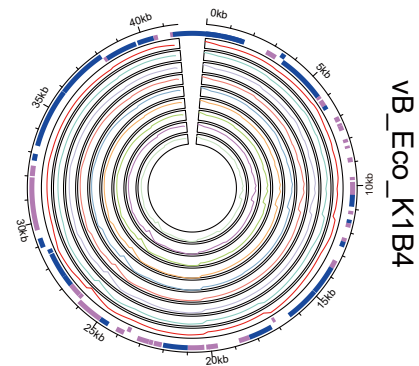

Supplement: Supplementary file 9 [file Image_6.pdf]
